# Vegetarian Diets and the Risk of Diabetes

**DOI:** 10.1007/s11892-018-1070-9

**Published:** 2018-09-18

**Authors:** Melissa D. Olfert, Rachel A. Wattick

**Affiliations:** Division of Animal and Nutritional Sciences, Davis College of Agriculture, Natural Resources, and Design, West Virginia University, G25 Agricultural Sciences Building, 1194 Evansdale Dr., Morgantown, WV 26506 USA

**Keywords:** Vegetarian, Diabetes, Vegan, Nutrition, Plant-based

## Abstract

**Purpose of Review:**

Worldwide, diabetes has increased steadily and in recent years, drastically. The majority of diabetes cases are type 2 (T2DM), caused by modifiable risk factors such as diet. Vegetarian diets have been studied over the past few decades for their preventative and therapeutic effects on diabetes and may be more beneficial than medication for diabetes management.

**Recent Findings:**

A vegetarian diet characterized by whole plant foods is most beneficial for diabetes prevention and management.

**Summary:**

Vegetarian diets are inversely associated with risk of developing diabetes independent of the positive association of meat consumption with diabetes development. Vegetarian diets range from vegan (no animal products), lacto-ovo-vegetarian (no animal meat, but consumes milk and eggs), pesco-vegetarian (consumes fish), and semi-vegetarian (occasional meat consumption). There has been an observed difference in the extent of preventative and therapeutic effects of these different types of diets. The most important aspect of any of these types of diets is emphasizing whole grains, fruits and vegetables, legumes, and nuts and reducing saturated and trans fats.

## Introduction

Diabetes has reached epidemic levels, with an estimated 451 million cases worldwide in 2017 [1]. This number is expected to grow to 693 million by 2045 [1]. The economic burden of this disease is large, with an estimated 850 billion USD spent on healthcare costs annually [1]. Preventing the onset of this disease and finding sustainable ways to manage it can have positive effects for the health of the population as well as the economy.

About 90% of diabetes diagnoses are type 2 (T2DM), which is lifestyle-related [2]. The causes of type 2 diabetes mellitus are largely modifiable, particularly diet. The functionality of certain foods has been found to improve the symptoms of diabetes [3]. Foods that are particularly therapeutic are whole grains, legumes, fruits and vegetables, and the compound found in many of these foods, polyphenols [3]. A Western diet is typically low in these foods and high in animal protein, saturated fat, and refined carbohydrates. As countries develop a more Westernized diet, their rates of diabetes increase [2]. A diet that differs from typical Western foods is a vegetarian one, and vegetarians in the US have a lower prevalence of diabetes than omnivores (consuming both plant and animal foods) [2, 4–6]. In addition, adoption of a vegetarian diet has been shown to be more beneficial in improving diabetes symptoms than traditional medication in some studies [7–10]. Because of the abundance of therapeutic foods mentioned above that vegetarians may be consuming more of and the lower prevalence of diabetes in vegetarians, studying this diet in preventing or managing diabetes is of interest to clinicians and healthcare professionals. There are multiple types of vegetarian diets, and determining the one that is most therapeutic and easiest for patients to adhere to is vital for clinical care. This review will discuss the important discoveries to date on the effects of vegetarian diets on preventing and treating diabetes, and examine which type of vegetarian diets may be most beneficial.

## Types of Vegetarian Diets

The term “vegetarian” encompasses a variety of types and degrees of food intake in diets. The most restrictive diet is vegan, consuming no foods from any animal. Lacto-ovo-vegetarians do not consume meat obtained from animal muscle, but still consume milk, milk products, and eggs on a regular basis. Pesco-vegetarians consume fish, milk, milk products, and eggs. Semi-vegetarians consume meat and meat products minimally but on a regular basis. Some studies have distinguished between these types of diets, and others have grouped them together.

## Vegetarian Diets in Preventing Diabetes

Research to date has looked at both the prevention of and treating of diabetes using a vegetarian diet. Many observational studies for this area of focus have followed Seventh-Day Adventists [4, 10], as they have had rates of any type of vegetarianism of about 50% and refrain from tobacco, caffeine, and alcohol, making their diet habits easier to isolate [11]. This population has only 45% the rate of diabetes of the general population [11]. Even small amounts of meat consumption have been shown to increase the risk of developing diabetes among this population. A study that examined 8401 adult Seventh-Day Adventists without diabetes at baseline found that at after 17 years of follow-up, those who consumed meat just once per week had a 29% higher risk of developing diabetes than those who refrained, and this risk increased to 38% if the meat was processed [5]. Lifelong adherence to a vegetarian diet in this population was associated with a 74% reduced risk of developing diabetes compared to a diet that included weekly meat consumption [5].

A recent observational study followed 2918 non-smoking, non-alcohol drinking Buddhists who were free of chronic disease at baseline [12•]. After a mean follow-up of 5 years, there were 183 cases of diabetes. Diet, fasting glucose, and HbA1c were measured throughout the study. Diet was measured using a food frequency questionnaire and did not distinguish between types of vegetarian diets. They found that a lifelong adherence to a vegetarian diet was associated with a 35% lower risk of developing diabetes. Importantly, those adopting a vegetarian diet after being non-vegetarian had a 53% lower risk for developing diabetes than non-vegetarians. These results were essentially unchanged after adjusting for other lifestyle factors such as age, physical activity, and family history of diabetes.

Another recent study using a sample of 6798 participants from the Rotterdam Study in the Netherlands examined the difference in associations of insulin resistance, pre-diabetes, and T2DM among plant- versus animal-based diets [13••]. Data on dietary intake, insulin resistance, presence of pre-diabetes, and presence of T2DM were collected. Adherence to a plant-based diet was measured by assigning participants a score on the plant-based dietary index using responses from food frequency questionnaire. They found that a higher score on the plant-based dietary index was associated with lower insulin resistance [*β* = − 0.09 (95% CI − 0.10 to − 0.08)], lower risk of pre-diabetes [HR = 0.89 (95% CI 0.81 to 0.98)], and lower risk of T2DM [HR = 0.82 (95% CI 0.73 to 0.92)] after adjusting for lifestyle and sociodemographic characteristics. Even after adjustment for BMI, these associations remained significant for insulin resistance [*β* = − 0.05 (95% CI − 0.06 to − 0.04)] and T2DM risk [HR = 0.87 (95% CI 0.79 to 0.99)].

A vegetarian diet adopted early on can prevent the onset of diabetes, but determination of the therapeutic effects on diabetes is also important for clinical practice and diabetes self-management.

## Vegetarian Diets in Diabetes Treatment

The impact of dietary interventions on diabetes has also been examined. Several studies have found that medication use significantly decreased when participants adopted any type of vegetarian or vegan diet. Vegan diet interventions began in 1999 with a 12-week study, where adult participants were recruited from the Georgetown Medical Center. Those adhering to a low-fat, vegan diet (*n* = 7) saw a 28% reduction in their fasting plasma glucose and significantly greater weight loss than those adhering to a traditional diabetes diet (*n* = 4) [14]. One study investigated the effects of a 16-day intervention with a low-fat, high-carbohydrate vegetarian diet in 20 men with type 2 diabetes [7]. All men were first fed a traditional diabetes diet for 7 days followed by a diet consisting of carbohydrates obtained from whole grains and plant foods for the rest of the study. While on the traditional diabetes diet, no changes were seen in insulin dosage, fasting plasma glucose, or urine glucose levels. After being put onto the low-fat, high-carbohydrate diet, nine of the men who had received 15 to 20 units of insulin per day no longer needed it and discontinued use. The remaining who had been on a higher insulin dosage decreased their use by more than half. However, mean fasting plasma glucose did not drop significantly. Barnard and colleagues found a significant drop in fasting plasma glucose after a 26-day near-vegetarian intervention consisting of high complex-carbohydrates, high fiber, low fat, and low salt. Thirty-nine percent of participants who were on medication at baseline discontinued use due to no longer needing insulin injections after adhering to the diet [8]. Another study used a randomized controlled trial to examine the effects of a vegan diet to the National Cholesterol Education Program (NCEP) diet in overweight adult women. Participants not only saw improvements in insulin sensitivity and weight loss, but a sustained weight loss at a 2-year follow-up, showing the potential of adhering to a diet that the population may view as restrictive [15]. When examining the effects of a vegan diet compared to the American Diabetic Association’s (ADA) recommended diet, significantly greater reductions in HbAlc levels (0.96 percentage point in vegan group versus 0.56 points in control group, *p* = 0.09) and body weight (6.5 kg in vegan group versus 3.1 kg in control group, *p* < 0.001) were found among those on the vegan diet, and these effects were sustained at a 1-year follow-up [9].

A study in 2010 by Kahleova et al. examined the effects of a vegetarian diet versus a conventional diabetic diet in managing type 2 diabetes [16]. In this 24-week randomized controlled trial, 74 patients who were randomly assigned to either a calorie-restricted vegetarian or a conventional diet, with meals provided. Patients were assessed at baseline, 12 weeks, and 24 weeks, with the second half of the study including aerobic exercise. At the conclusion of the trial, 43% of those adhering to the vegetarian diet were able to reduce medication use compared to just 5% in the control group. Those in vegetarian diet arm of the trial also had greater body weight reduction, greater insulin sensitivity, and greater subcutaneous and visceral fat loss. These findings were even stronger after accounting for the addition of exercise in the second half of the study.

In another study by Kahleova et al., using the same diabetic population as above, the psychological effects of adopting a vegetarian diet was examined [17]. The investigators assessed quality of life, eating behavior, and depressive symptoms. They found an increase in quality of life and decrease in depressive symptoms in both groups from baseline to week 12, but in weeks 12–24, only in the vegetarian group were further improvements observed. For dietary restraint, there were increases in both groups from baseline to 12 weeks, with greater increases in the control group, and no change from weeks 12–24. This study showed that adopting a vegetarian diet has both physical benefits and psychological benefits for type 2 diabetes patients.

## Vegetarian Diets and Reducing Diabetes Complications

An important component of diabetes management is reducing cardiovascular disease (CVD) risk, as those with diabetes have a 2–4 times greater risk of suffering from CVD [18]. A cross-sectional study found that those who adhered to a lacto-ovo-vegetarian diet had significantly decreased CVD risk factors, specifically blood pressure, serum cholesterol, and blood glucose levels than those adhering to an omnivorous diet [19]. Another study that examined ischemic heart disease risk of vegetarians versus non-vegetarians in a large British sample of 44,561 individuals found that vegetarians had a lower BMI, non-HDL cholesterol, and systolic blood pressure than the non-vegetarians. Importantly, vegetarians had a 32% lower risk of ischemic heart disease [20].

When looking at other diabetes risk factors and comorbidities, a recent study found that those adhering to a vegan diet supplemented with B12 had a significantly larger decrease in neuropathy pain than the control group receiving just B12 supplementation, with a difference of 9.1 point reduction in those consuming the vegan diet versus a 0.9 point reduction in the control group on the pain scale [21]. A study examining patients who had diabetic neuropathy and renal failure who followed a vegan diet found significant improvements in creatinine clearance (1.48 to 0.13 mL/min), urine protein levels (5.2 to 2.8 g/day), cholesterol levels (254 to 165 mg/dL), and blood glucose levels (166 to 131 mg/dL) after about 1 year on the vegan diet [22].

## Differences in Diabetes Prevalence and Risk by Types of Vegetarian Diets

As mentioned previously, there are different types, combinations, and degrees of vegetarian diets: vegan, lacto-vegetarian, ovo-vegetarian, pesco-vegetarian, and semi-vegetarian. Studies have found that among the varying vegetarian diets, there are different therapeutic effects.

Using data from the Adventist Health Study-2, a 2009 study by Tonstad and colleagues investigated the relationship of vegetarian diet with the prevalence of diabetes, distinguishing between types of vegetarian diets [10]. They used self-reported data on weight, lifestyle, and food frequency questionnaires to categorize type of vegetarian diets. Participants were designated as vegan, lacto-ovo-vegetarian, pesco-vegetarian, semi-vegetarian, and non-vegetarians. They found that the prevalence of diabetes increased incrementally across these groups, from vegans having the lowest (2.9%), followed by lacto-ovo-vegetarians (3.2%), pesco-vegetarians (4.8%), semi-vegetarians (6.1%), and non-vegetarians (7.6%).

The differences in risk by vegetarian diet type bring to light an important factor in determining the health benefits of vegetarian diets. The adoption of vegetarian diets is increasing worldwide; however, not every vegetarian diet follows the health principles that are beneficial, such as consuming whole grains, vegetables, fruits, and legumes. To examine the differences in type 2 diabetes risk of vegetarians who consume an unhealthy diet (characterized by refined grains, starchy foods, added sugars, low fruits and vegetables) or healthy diet (characterized by whole grains, fruits, vegetable, legumes), Satija and colleagues examined data from three different cohorts, the Nurses’ Health Study, the Nurses’ Health Study 2, and the Health Professionals Follow-up Study [23••]. Diet data collected every 2 to 4 years was used to create a Plant-Based Diet Index (PDI), assigning positive scores to plant foods and reverse scores to animal foods. A healthful Plant-Based Diet Index (hPDI) and Unhealthy Plant-Based Diet Index (uPDI) that distinguished between healthy and unhealthy plant foods were also created. The hPDI assigned positive scores to whole grains, fruits, vegetables, nuts, vegetable oils, tea, and coffee and reverse scores to fruit juices, sweetened beverages, refined grains, potatoes, sweets, desserts, and animal foods while the uPDI used the opposite approach. They found that the PDI and hPDI were inversely associated with T2DM, and the uPDI was positively associated with T2DM. This shows the benefit of following a vegetarian diet that is high in whole grains, vegetables, fruits, nuts, and legumes in preventing T2DM.

## Mechanisms of Vegetarian Diet on Diabetes Risk Reduction

The differences in therapeutic effects of the various vegetarian diets may be explained by examining what types of foods are beneficial for diabetes prevention and management.

### Increased Fiber, Fruit, and Vegetable Intake

Replacing meat with other types of protein foods may incorporate more beneficial nutrients into the diet. For example, soybeans are a common protein substitution for lacto-ovo-vegetarians and vegans. This food is high in lysine, leucine, isoleucine, phenylalanine, calcium, and phosphate, all of which have been shown to aid in increasing glycemic control and insulin sensitivity [12•, 24]. Overall, vegetarians have a higher intake of fruits and vegetables, fiber, and antioxidants, and phytochemicals. There is evidence that high consumption of fruits and vegetables can decrease the risk of developing T2DM [3, 25•]. The high amounts of soluble fiber in the diet may be beneficial for diabetes management, as soluble fiber binds glucose, slowing absorption into the blood [25•]. Higher intake of whole grains and vegetables have been found in vegetarians compared to non-vegetarians [12•], and these foods have high amounts of fiber and magnesium. Whole grains have been found to reduce the risk of developing diabetes [25•]. Consuming sufficient amounts of magnesium is important, as a deficiency can possibly impair insulin signaling [12•].

### Weight Control

Plant foods have higher amounts of fiber and lower caloric content, aiding in weight control and reducing diabetes risk [23••]. Weight loss aids in insulin sensitivity and glycemic control [11]. Even in studies that investigated the effects of a vegetarian diet and did not see weight loss showed improvements in insulin sensitivity and glycemic control [7], showing that there are other mechanisms of the diet that are therapeutic beyond weight control.

### Reduced Saturated Fat Intake

Vegetarians also consume less saturated fats, and replacing these fats with polyunsaturated fatty acids has been shown to be beneficial for diabetes and its comorbidities [3]. Semi-vegetarians and pesco-vegetarians have shown to have less protection against diabetes than those on more animal product restrictive vegetarian diets. Semi-vegetarians who consume meat minimally but regularly have an intake of saturated fats, which lowers insulin secretion and can trigger beta-cell apoptosis [12•]. There is evidence that omega-3 fatty acids from fish may decrease insulin secretion [12•]. This may explain why reliance on seafood for protein by pesco-vegetarians may counteract the beneficial effects of the other foods they are consuming and lessen the therapeutic effects of not consuming meat.

Meat consumption has been shown to increase risk of diabetes and its comorbidities. Recent publications have suggested the addition of red meat to the diabetes risk factors list [26]. A 2011 study examined three cohorts for the association between unprocessed and processed red meat consumption and incidence of T2DM in US adults [27]. They found that in all three cohorts, there was a positive association between T2DM risk and meat consumption, even after adjusting for other lifestyle factors. This is consistent with previous studies finding that even just consuming meat once a week increases risk for diabetes [5].

In summary, vegetarian diets have high amounts of fiber, vitamins, minerals, antioxidants, polyphenols, and phytochemicals, and low amounts of saturated fats and trans fats. In addition, the protein consumed by vegetarians commonly contains fiber and high amounts of beneficial vitamins and minerals. Characteristics of an unhealthy vegetarian diet would include added sugars and refined grains, and thus emphasizing incorporation of whole grains into the diet is an important component when prescribing a vegetarian diet.

## Conclusion

The benefits of all types of vegetarian diets in the prevention and treatment of diabetes have been well established. Clinicians and healthcare providers should feel confident in recommending a vegetarian diet to their patients who have pre-diabetes or T2DM. However, the type of foods that should be consumed while following this diet is critical to achieve the therapeutic effects. As Satija et al. demonstrated, a vegetarian diet that is high in unhealthy foods such as refined grains, saturated fats, and added sugars is positively associated with T2DM compared to a vegetarian diet with lower amounts of these nutrients. The foods that are important to consume while following a vegetarian diet for treating diabetes are whole grains, fruits, vegetables, nuts, legumes, and unsaturated fats. Each of these foods has functional components that reduce the symptoms of diabetes. For these reasons, clinician knowledge and patient education is extremely important to ensure the adherence to a healthy vegetarian diet. No matter the type of vegetarian diet followed, there are therapeutic effects. However, there is evidence that a vegan diet has the most benefits for reducing the fasting plasma glucose levels of persons with diabetes and other complications, such as CVD risk. Patients should follow the diet that they feel they can adhere to best.

Future research is needed to examine intervention trials adopting different variations of a vegetarian diet to further assess which type, combination, or degree is most beneficial. Intervention trials should incorporate long-term follow-up to measure adherence of different types of vegetarian diets, as this is important for patient recommendations.
